# Are There Gender Differences in the Benefits of Multidisciplinary Care in Patients with Heart Failure? Results from the UMIPIC Program

**DOI:** 10.3390/jcm14165818

**Published:** 2025-08-17

**Authors:** Alicia Conde-Martel, Manuel Méndez-Bailón, Manuel Montero-Pérez-Barquero, Álvaro González-Franco, José Manuel Cerqueiro, José Pérez-Silvestre, José María Fernández-Rodríguez, Pau Llàcer, Jesús Casado, Francesc Formiga, Prado Salamanca-Bautista, Jose Carlos Arévalo-Lorido, Luis Manzano

**Affiliations:** 1 Internal Medicine Department, Hospital Universitario de Gran Canaria Dr. Negrín, Las Palmas de Gran Canaria, 35010 Las Palmas, Spain; 2 Health Sciences Faculty, Universidad de Las Palmas de Gran Canaria, 35016 Las Palmas, Spain; 3 Internal Medicine Department, Hospital Clínico San Carlos, Instituto de Investigación Sanitaria del Hospital Clinico San Carlos (IdISSC), 28040 Madrid, Spain; manuelmenba@hotmail.com; 4 Faculty of Medicine, Universidad Complutense, 28040 Madrid, Spain; 5 Internal Medicine Department, Hospital Universitario Reina Sofía, Instituto de Investigación Biomédica de Cordoba (IMIBIC), 14004 Cordoba, Spain; montero.manolo@gmail.com; 6 Internal Medicine Department, Hospital Universitario Central de Asturias, 33011 Oviedo, Spain; 7 Internal Medicine Department, Hospital Universitario Lucus Augusti, 27003 Lugo, Spain; 8 Internal Medicine Department, Consorcio Hospital General Universitario de Valencia, 46014 Valencia, Spain; 9 Internal Medicine Department, Hospital Carmen y Severo Ochoa, 33819 Cangas del Narcea, Spain; chemachir@hotmail.com; 10 Internal Medicine Department, Hospital Universitario Ramón y Cajal, Instituto Ramón y Cajal de Investigación Sanitaria (IRYCIS), Universidad de Alcalá, 28801 Madrid, Spain; 11 Internal Medicine Department, Hospital Universitario de Getafe, 28905 Madrid, Spain; 12 Department of Medicine, Faculty of Medicine, Health and Sports, 28670 Madrid, Spain; 13 Internal Medicine Department, Hospital Universitario de Bellvitge, L’Hospitalet de Llobregat, 08907 Barcelona, Spain; 14 Internal Medicine Department, Hospital Universitario Virgen Macarena, 41009 Sevilla, Spain; 15 Faculty of Medicine. Universidad de Sevilla, 41009 Sevilla, Spain; 16 FundeSalud, 06800 Mérida, Spain; joscarlor@gmail.com

**Keywords:** heart failure, sex, UMIPIC, clinical outcomes

## Abstract

**Background/Objectives**: Heart failure (HF) is a leading cause of hospitalization in older adults, with significant sex differences in presentation, treatment, and outcomes. Transitional care models may benefit women more, yet they often receive less follow-up. This study assessed whether the clinical impact of the UMIPIC multidisciplinary HF management program differs by sex. **Methods:** This prospective, multicenter, observational cohort study included HF patients enrolled in the UMIPIC program or followed through conventional care in the RICA registry. Outcomes (30-day and one-year mortality and readmissions) were compared between groups, stratified by sex. Multivariate Cox models adjusted for age, HF phenotype, comorbidities, and baseline therapy. **Results:** A total of 5644 HF patients were included, with 2034 (36%) managed in UMIPIC and 3610 (64%) receiving conventional care. Women represented 55% of UMIPIC patients and were older, with higher prevalence of hypertension, anemia, and HF with preserved ejection fraction (HFpEF) compared to conventional care. At 30 days, women in UMIPIC had lower all-cause mortality (4.0% vs. 8.0%), cardiovascular mortality (2.0% vs. 6.0%), and readmissions (9.0% vs. 18.0%; all *p* < 0.01); these benefits persisted at one year. In multivariate analysis, UMIPIC enrollment remained protective (HR: 0.79; 95% CI: 0.71–0.87; *p* < 0.001). In men, UMIPIC patients were older with more comorbidities and higher HFpEF prevalence. They also showed lower 30-day mortality (2.0% vs. 8.0%; *p* < 0.05) and readmissions (8.0% vs. 18.0%; *p* < 0.01), with benefits maintained at one year. UMIPIC enrollment remained independently associated with reduced one-year mortality in men (HR: 0.79; 95% CI: 0.71–0.88; *p* < 0.001). **Conclusions:** The UMIPIC multidisciplinary care model reduced one-year mortality and readmissions in both women and men with HF, supporting integrated care strategies to improve outcomes in this high-risk population.

## 1. Introduction

Heart failure (HF) is a clinical syndrome and the leading cause of hospitalization in patients over 65 years of age [1]. Its prevalence increases markedly with age, making it particularly prevalent in older adults. The burden of HF continues to rise, posing a substantial social and economic challenge to healthcare systems worldwide [2]. In recent years, the management of HF has become increasingly complex due to the introduction of novel pharmacological therapies, cardiac devices, and interventional strategies. Moreover, most patients present with multiple comorbidities, further complicating clinical management.

To reduce hospital admissions and mortality, current HF clinical practice guidelines strongly recommend the implementation of multidisciplinary HF management programs [3,4]. The UMIPIC model (Comprehensive Management Unit for Patients with Heart Failure) provides structured, multidisciplinary, outpatient care and has demonstrated reductions in hospital admissions and mortality [5].

Over the past decade, increasing efforts have been made to understand sex-specific differences in HF. Well-documented differences exist between men and women in terms of pathophysiology, clinical presentation, prognosis, and treatment [6,7,8,9,10,11]. Women have been historically underrepresented in clinical trials, tend to report more symptoms, and experience a poorer quality of life [12]. They also receive fewer guideline-recommended therapies [13], including lower referral rates to specialist care, device therapies, and heart transplantation [14]. Nevertheless, women with HF generally exhibit lower mortality rates compared to men [12,15].

The clinical trajectory and healthcare resource utilization following hospitalization for HF may also differ by sex. Although the available literature is limited, existing evidence suggests that patient-centered transitional care models confer greater clinical benefit to women than to men after HF hospitalization [16]. However, it has been reported that women—particularly those over the age of 75—receive less outpatient follow-up, experience suboptimal medication titration, and have reduced access to optimal diagnostic and therapeutic interventions compared to men [17]. These disparities highlight the need for targeted strategies to address and close sex-related gaps in HF care. Despite the demonstrated benefits of the UMIPIC model in HF management, data assessing whether its clinical impact differs by sex remain limited.

The objective of this study is to analyze sex-based differences and their prognostic impact on one-year mortality and hospital readmission rates in patients with HF managed through the UMIPIC multidisciplinary care model, compared to those receiving conventional follow-up.

## 2. Materials and Methods

### 2.1. Study Design and Population

This was a prospective, multicenter, observational cohort study that included patients diagnosed with HF who were either enrolled in the UMIPIC multidisciplinary management program or followed through conventional care as recorded in the RICA (National Registry of Heart Failure) database [5]. The registry includes data from 52 Spanish hospitals. All patients consecutively admitted to Internal Medicine units with acute HF and seen by physicians participating in the registry were eligible for inclusion. Eligible patients met the following criteria: hospitalization due to HF, either as a first episode or as a decompensation of previously diagnosed chronic HF. Exclusion criteria included the need for specialized cardiological interventions (e.g., ischemic procedures requiring catheterization, device implantation, valve replacement, or pending cardiac transplantation). Additionally, patients with functional and cognitive impairments lacking adequate social or familial support were also excluded.

The type of follow-up after hospital discharge was non-randomized and based on routine clinical practice. All patients were followed for a minimum of one year. Patients were categorized into two groups based on the type of post-discharge follow-up:

(1) UMIPIC group: Patients enrolled in the Comprehensive Management Unit for Patients with Heart Failure (UMIPIC), receiving structured, multidisciplinary outpatient care. The UMIPIC program is a protocol-driven model designed for older patients with chronic HF and multiple comorbidities, delivered in outpatient settings by internists and specialized nurses. It is based on five core components [5]: (1) comprehensive management of HF and comorbidities, (2) continuous follow-up through in-person and telephone visits, (3) structured education of patients and caregivers to promote adherence, self-care, and early recognition of symptoms, (4) rapid access to medical attention for acute decompensations, and (5) coordination with other specialists when needed. Care is individualized and follows clinical practice guidelines, including pharmacological optimization, lifestyle counseling, and functional monitoring. Inclusion in the UMIPIC program was based on a high risk of early readmission, assessed through the presence of recurrent hospitalizations or emergency visits in the previous year, poor clinical status at discharge (including renal dysfunction and high diuretic requirements), or need for drug titration. A minimum level of cognitive and functional capacity, or the presence of a caregiver, was required to ensure adherence to the intensive follow-up protocol.

(2) Conventional care group (non-UMIPIC): Patients followed under usual care, without a structured HF management program.

Within each group, patients were further stratified by sex to assess gender-related differences.

### 2.2. Data Collection

Data were collected in an anonymized manner via a dedicated web platform (https://www.registrorica.org). (https://www.fesemi.org/grupos/cardiaca/proyectos/registro-rica) (accessed on 7 June 2022). Data coordination was overseen by the Heart Failure and Atrial Fibrillation Working Group of the Spanish Society of Internal Medicine (SEMI). Variables collected at discharge included demographic data, clinical characteristics (HF etiology, phenotype, comorbidities, NYHA functional class, and ejection fraction), and baseline treatments. Follow-up data encompassed all-cause mortality, cardiovascular mortality, and hospital readmissions at 30 days and one year. The primary outcomes were all-cause mortality and all-cause hospital readmissions at 30 days and one year post-discharge. Secondary outcomes included cardiovascular mortality and HF readmissions at both time points. Sex-based analyses were performed to explore differences in outcomes between women and men.

### 2.3. Statistical Analysis

Continuous variables were expressed as mean and standard deviation or median [interquartile range], as appropriate. Categorical variables were summarized as frequencies and percentages. Comparisons between groups were performed using Student’s *t*-test or Mann–Whitney U test for continuous variables and the chi-square test for categorical variables. Kaplan–Meier survival analysis and log-rank tests were used to evaluate time-to-event outcomes. To assess the association between sex, type of follow-up (UMIPIC vs. conventional care), and one-year clinical outcomes, multivariate Cox proportional hazards models were constructed, adjusting for potential confounders including age, HF phenotype, comorbidities, and baseline therapy. Covariates were selected based on clinical relevance and statistical significance in univariate analyses. In women, the model was adjusted for age, anemia, nursing home residence, hypertension, ischemic cardiomyopathy, left ventricular ejection fraction (LVEF) > 50%, and beta-blocker use. In men, the model included age, anemia, nursing home residence, hypertension, atrial fibrillation, LVEF > 50%, neoplasm, use of ACE inhibitors or ARBs, thiazide diuretics, and the Barthel Index. A *p*-value < 0.05 was considered statistically significant. Statistical analyses were performed using SPSS (Statistical Package for the Social Sciences, IBM Corp, Version 29.0, Armonk, NY, USA).

### 2.4. Ethical Considerations

The study was conducted in accordance with the Declaration of Helsinki and was approved by the Clinical Research Ethics Committee of Hospital Universitario Reina Sofía de Córdoba. Informed consent was obtained from all participants prior to their inclusion in the study.

## 3. Results

**Figure 1:**
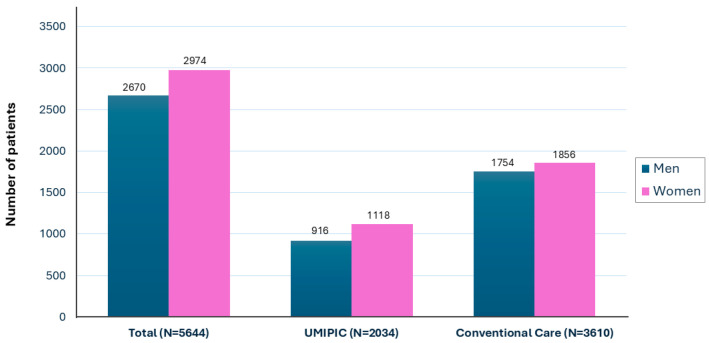
Sex distribution in total cohort and by type of follow-up.

A total of 5644 patients with HF were included in the study. Of these, 2034 patients (36%) were managed through the UMIPIC multidisciplinary care model, while 3610 (64%) received conventional follow-up as recorded in the RICA registry. In the overall cohort, 52.7% were women (*n* = 2974), and in the UMIPIC group, 55% were women (*n* = 1118) (Figure 1). The proportion of women included in the UMIPIC program was significantly higher than in the group receiving conventional follow-up (55% vs. 51%; *p* = 0.01).

### 3.1. Characteristics of Women

#### 3.1.1. Baseline Characteristics of Women in the UMIPIC and Conventional Care Groups

As shown in Table 1, women enrolled in the UMIPIC multidisciplinary care program were significantly older than those receiving conventional care (83.4 vs. 79.9 years; *p* < 0.001). Despite their older age, a lower proportion of UMIPIC patients resided in nursing homes (6.0% vs. 8.0%; *p* = 0.002).

Comorbid conditions such as hypertension, anemia, and dyslipidemia were more prevalent in the UMIPIC group, whereas ischemic heart disease was less frequent. Although physical function, as measured by the Barthel Index, was slightly lower in the UMIPIC group (76.2 vs. 78.6; *p* = 0.01), cognitive performance was better, as indicated by a lower score on the Pfeiffer test (1.6 vs. 2.1; *p* < 0.001).

A greater proportion of women in the UMIPIC group had HF with preserved ejection fraction (HFpEF, defined as LVEF >50%) compared to the conventional care group (69.8% vs. 64.5%; *p* = 0.004). Hypertensive etiology was also more common (53.7% vs. 41.6%; *p* < 0.001). When analyzed by individual NYHA classes, women in the UMIPIC group had a lower proportion in class I and slightly higher proportions in classes II and III, with similar representation in class IV compared with conventional care (*p* < 0.001)

Biomarker data showed higher NT-proBNP levels in the UMIPIC group (median 4119 vs. 3153 pg/mL; *p* = 0.004), suggesting greater clinical congestion.

Regarding pharmacological treatment, the use of beta-blockers, aldosterone antagonists, and thiazide diuretics was significantly more frequent in the UMIPIC group. Notably, direct oral anticoagulant (DOAC) use was more than twice as common in UMIPIC women compared to those receiving conventional care (15.1% vs. 7.2%; *p* < 0.001).

#### 3.1.2. Outcomes of Women with Heart Failure According to Care Setting

As shown in Table 2, women with HF who were managed within the UMIPIC multidisciplinary care model experienced significantly better clinical outcomes compared to those receiving conventional care.

At 30 days, the UMIPIC group exhibited lower rates of adverse events. All-cause mortality was significantly reduced (4.0% vs. 8.0%; RR: 0.50; 95% CI: 0.31–0.82; *p* < 0.01), as was cardiovascular mortality (2.0% vs. 6.0%; RR: 0.33; 95% CI: 0.12–0.56; *p* < 0.01). Hospital readmissions were also significantly lower in the UMIPIC group, both for all causes (9.0% vs. 18.0%; RR: 0.50; 95% CI: 0.39–0.63; *p* < 0.01) and specifically for HF (7.0% vs. 13.0%; RR: 0.54; 95% CI: 0.41–0.70; *p* < 0.01).

**Figure 2:**
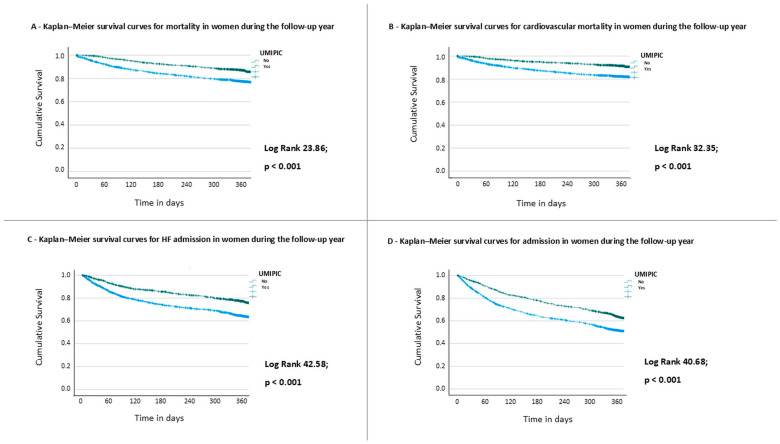
Outcomes during the follow-up in women. Kaplan–Meier curves for (**A**) all-cause mortality; (**B**) cardiovascular mortality; (**C**) heart failure admission; and (**D**) any-cause admission, during the one-year follow-up.

At one-year follow-up, the clinical benefits of the UMIPIC model were maintained. All-cause mortality remained significantly lower compared to conventional care (32.0% vs. 40.0%; RR: 0.80; 95% CI: 0.70–0.91; *p* < 0.01), as did cardiovascular mortality (21.9% vs. 30.0%; RR: 0.70; 95% CI: 0.59–0.83; *p* < 0.01). Similarly, the rates of hospital readmissions were substantially lower in the UMIPIC group, both for any cause (35.0% vs. 56.0%; RR: 0.62; 95% CI: 0.57–0.69; *p* < 0.01) and for heart failure-related admissions (30.0% vs. 42.0%; RR: 0.71; 95% CI: 0.63–0.81; *p* < 0.01). Kaplan–Meier survival curves are presented in Figure 2.

#### 3.1.3. Risk Factors for One-Year Mortality in Women with Heart Failure

In the univariate Cox analysis (Table 3), enrollment in the UMIPIC program was significantly associated with lower one-year mortality in women (HR: 0.79; 95% CI: 0.71–0.88; *p* < 0.001). Older age was associated with increased mortality risk (HR: 1.01 per year; 95% CI: 1.01–1.02; *p* < 0.001). Use of beta-blockers was associated with lower mortality (HR: 0.88; 95% CI: 0.80–0.98; *p* = 0.018).

In the multivariate Cox analysis (Table 3), the protective effect of UMIPIC remained significant (HR: 0.79; 95% CI: 0.71–0.87; *p* < 0.001). Beta-blocker use was also independently associated with lower mortality (HR: 0.90; 95% CI: 0.81–0.99; *p* = 0.032), whereas age was associated with an increased risk of mortality (HR: 1.01; 95% CI: 1.01–1.02; *p* < 0.001). Kaplan–Meier survival curves for women (Supplementary Figure S1) demonstrate a significantly higher one-year survival in the UMIPIC group compared with non-UMIPIC care across all NYHA functional classes (log-rank *p* < 0.001). The survival benefit was accompanied by a marked attenuation of mortality differences between advanced (NYHA III–IV) and milder (NYHA I–II) functional classes in UMIPIC-treated patients.

### 3.2. Characteristics of Men

#### 3.2.1. Baseline Characteristics of Men in the UMIPIC and Conventional Care Groups

Men managed within the UMIPIC multidisciplinary care model were significantly older than those receiving conventional care (mean age 80.9 vs. 76.5 years; *p* < 0.001). Despite their older age, the proportion of nursing home residents was markedly lower in the UMIPIC group (4.7% vs. 10.4%; *p* < 0.001). (Table 4).

Functional status, as measured by the Barthel Index, was slightly lower in the UMIPIC group (84.8 vs. 87.8; *p* < 0.001), indicating marginally reduced physical function. However, cognitive performance, assessed with the Pfeiffer test, was slightly better in UMIPIC patients (1.1 vs. 1.3; *p* = 0.008), suggesting preserved cognitive function.

Several comorbidities were more prevalent in the UMIPIC group, including hypertension, atrial fibrillation, neoplasms, and anemia. In contrast, ischemic heart disease was less frequent among UMIPIC patients. No significant differences were observed in the prevalence of diabetes, chronic kidney disease, stroke, chronic obstructive pulmonary disease, or liver disease.

HFpEF was more common among UMIPIC patients (48.4% vs. 42.0%; *p* = 0.002), as was hypertensive etiology (37.4% vs. 26.9%; *p* < 0.001). In men, UMIPIC patients had fewer in NYHA class I, more in class III, and similar proportions in classes II and IV (*p* < 0.01). Regarding pharmacological management, UMIPIC patients were more frequently treated with aldosterone antagonists, loop diuretics, thiazide diuretics, and direct oral anticoagulants (DOACs) (14.7% vs. 7.5%; *p* < 0.001). In contrast, the use of angiotensin-converting enzyme inhibitors (ACEi) or angiotensin receptor blockers (ARBs) was significantly lower in the UMIPIC group (56.0% vs. 64.7%; *p* < 0.001).

#### 3.2.2. Outcomes of Men with Heart Failure According to Care Setting

Men with HF managed within the UMIPIC multidisciplinary care model experienced significantly better outcomes compared to those receiving conventional care, both in the short and long term (Table 5). At 30 days, all-cause mortality was significantly lower in the UMIPIC group (2.0% vs. 8.0%; RR: 0.25; 95% CI: 0.16–0.40; *p* < 0.05), as was cardiovascular mortality (2.0% vs. 5.0%; RR: 0.40; 95% CI: 0.23–0.70; *p* < 0.001). Readmission rates were also substantially reduced among UMIPIC patients, both for all causes (8.0% vs. 18.0%; RR: 0.44; 95% CI: 0.34–0.58; *p* < 0.01) and for heart failure-related causes (5.0% vs. 11.0%; RR: 0.45; 95% CI: 0.32–0.64; *p* < 0.01).

**Figure 3:**
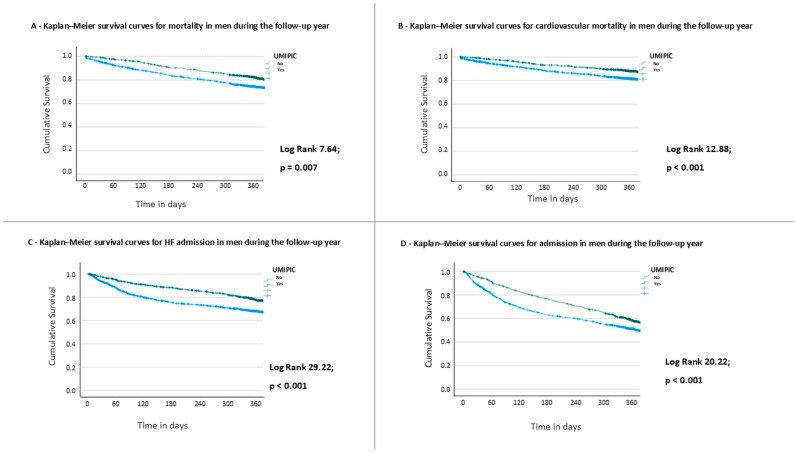
Outcomes during the follow-up in men. Kaplan–Meier curves for (**A**) all-cause mortality; (**B**) cardiovascular mortality; (**C**) heart failure admission; and (**D**) any-cause admission, during the one-year follow-up.

At one year, these favorable trends persisted. All-cause mortality remained lower in the UMIPIC group (37.0% vs. 41.0%; 0.95; 95% CI: 0.84–1.00; *p* < 0.05), as did cardiovascular mortality (23.0% vs. 30.0%; RR: 0.77; 95% CI: 0.64–0.92; *p* < 0.01). Similarly, one-year readmission rates were significantly reduced in UMIPIC patients for both all-cause (50.0% vs. 58.0%; RR: 0.86; 95% CI: 0.79–0.94; *p* < 0.01) and heart failure-specific readmissions (29.0% vs. 38.0%; RR: 0.76; 95% CI: 0.64–0.89; *p* < 0.01). Kaplan–Meier survival curves are presented in Figure 3.

#### 3.2.3. Risk Factors for One-Year Mortality in Men with Heart Failure

In the univariate Cox analysis (Table 6), enrollment in the UMIPIC program was significantly associated with lower one-year mortality (HR: 0.782; 95% CI: 0.701–0.873; *p* < 0.001). The presence of a neoplasm (HR: 1.159; 95% CI: 1.003–1.320; *p* = 0.046), use of thiazide diuretics (HR: 1.174; 95% CI: 1.026–1.342; *p* = 0.019), and lower Barthel Index scores (HR: 0.99; 95% CI: 0.988–0.992; *p* < 0.001) were also significantly associated with one-year mortality.

In the multivariate Cox analysis (Table 6), the protective effect of UMIPIC remained significant (HR: 0.79; 95% CI: 0.71–0.88; *p* < 0.001). Additionally, both the presence of a neoplasm (HR: 1.15; 95% CI: 1.01–1.32; *p* = 0.027) and thiazide diuretic use (HR: 1.16; 95% CI: 1.02–1.33; *p* = 0.027) remained independently associated with increased mortality, while a higher Barthel Index continued to be protective (HR: 0.990; 95% CI: 0.988–0.993; *p* < 0.001). Kaplan–Meier survival curves for men (Supplementary Figure S2) show a significantly higher one-year survival in the UMIPIC group compared with non-UMIPIC care across all NYHA functional classes (log-rank *p* < 0.001). The survival benefit was particularly evident in patients with advanced functional class (NYHA III–IV), with attenuation of the steep early mortality decline observed in the non-UMIPIC group.

## 4. Discussion

This study demonstrates that multidisciplinary care through the UMIPIC program is associated with significant improvements in both short- and long-term outcomes for patients with HF, with beneficial effects observed in both men and women.

Prior studies suggest that females experience disparities in care, including delayed diagnoses and gaps in evidence-based therapies [6,7]. Also, previous studies have shown that male patients received more specialist and multidisciplinary clinic care [18,19].

In our study, the proportion of women included in the UMIPIC program was significantly higher than in the group receiving conventional follow-up. This contrasts with findings from other healthcare settings, where women are often underrepresented in multidisciplinary HF programs [19]. Interestingly, the proportion of men referred to the UMIPIC program was slightly lower than women. This pattern may reflect the internist-led approach of the UMIPIC model, which emphasizes a comprehensive assessment of clinical complexity and comorbidities.

Given the similar risk of rehospitalization described in both women and men [20], referring a high proportion of women to multidisciplinary programs seems appropriate. This contrasts with previous reports where women are often underrepresented [18]. Prioritizing inclusion based on global clinical risk rather than sex alone could have contributed to a more equitable referral process, representing a potential step forward in addressing gender disparities in HF care.

Moreover, the high proportion of patients with HFpEF, a phenotype more prevalent among women [6,12,15], could have influenced the observed referral profile. Until recently, there were no disease-modifying treatments available for this population, and comorbidity management remained the cornerstone of care [3].

Patients included in the UMIPIC program, both men and women, exhibited distinct clinical characteristics compared to those receiving conventional follow-up. They were older and had a higher comorbidity burden, with hypertension and anemia being more prevalent in both sexes, while men more commonly had atrial fibrillation, neoplasms and a higher Charlson Comorbidity Index, differences that have been described previously [21]. Additionally, patients in the UMIPIC program more frequently had HF of hypertensive etiology and preserved ejection fraction, although this phenotype was more common in women, as is well known [6,12,15]. On the other hand, cognitive impairment was less frequent, possibly indicating a selection bias toward patients capable of participating actively in the program.

Interestingly, NT-proBNP levels were higher in women followed within UMIPIC, but not in men. Greater congestion and more pronounced symptoms in women have been described [20]. The higher NT-proBNP concentrations observed in women enrolled in the UMIPIC program may indicate more severe clinical congestion and greater baseline risk. Nevertheless, their outcomes were better than those of women receiving conventional care. This dissociation between biomarker levels and clinical improvement supports the effectiveness of the multidisciplinary approach even in higher-risk patients and could translate into an even greater benefit of the program in this subgroup.

Regarding treatment, in the UMIPIC group, both men and women more frequently received ACE inhibitors and/or ARBs, mineralocorticoid receptor antagonists, thiazide diuretics, and DOACs. More than two-thirds of women had HFpEF, and the high prevalence of hypertension along with a likely greater degree of congestion justifies the use of more intensive combination therapy. Statin use was also more common among women, probably due to their higher prevalence of dyslipidemia. In men, approximately half of whom had HFpEF, the use of these treatments as well as loop diuretics was also more frequent in the UMIPIC group.

Regarding outcomes, the UMIPIC program was associated with a significant reduction in mortality, consistently observed in both men and women, at both 30 days and one year, as well as a decrease in early (30-day) and one-year hospital readmissions. This effect persisted after adjusting for other variables. Although this benefit has been reported before [22], the differences between gender have not been analyzed in detail.

These results suggest that both men and women benefit from receiving care in the UMIPIC, although the degree of mortality reduction may vary by sex. The data indicate that access to multidisciplinary follow-up in UMIPIC has a favorable impact on clinical outcomes, as reflected in the lower mortality rates compared to those who do not receive these specialized interventions.

Overall, these findings reinforce the importance of UMIPIC in reducing adverse events for both men and women, emphasizing the need to continue exploring potential sex-related disparities in access and treatment response in order to promote equity in care.

This study has several limitations. First, it is an observational, non-randomized study, which limits the ability to establish causal relationships. Data were obtained from Spanish hospitals and a specific program (UMIPIC), which may limit extrapolation to other countries or healthcare settings. Additionally, data were recorded from clinical registries, and some relevant variables may be missing. Detailed information on medication dosing and optimization was not evaluated, and comprehensive data on device implantation were also not collected. Although UMIPIC includes older, multimorbid, and frail patients, it excludes those with severe cognitive or functional impairment or insufficient social support. This may have introduced a selection bias toward more autonomous and adherent patients, limiting the generalizability of our findings to the most severely impaired.

Nevertheless, this study provides relevant information. Importantly, it is a real-world study that offers valuable insight into the clinical profile of patients referred for multidisciplinary management. It reflects everyday clinical practice and highlights how comprehensive programs may be preferentially offered to patients with specific characteristics, such as advanced age, multiple comorbidities, or HFpEF, particularly among women.

## 5. Conclusions

These findings suggest that the UMIPIC multidisciplinary care model is associated with improved short- and long-term outcomes in both women and men with heart failure, reducing mortality and hospital readmissions at one-year follow-up. This highlights the importance of specialized and integrated care strategies for all patients with heart failure. The results reinforce the effectiveness of the UMIPIC model in improving clinical outcomes and reducing healthcare utilization, particularly among older patients with a high burden of comorbidities.

## Figures and Tables

**Table 1:** Baseline characteristics of heart failure women from RICA (total) and followed in UMIPIC and non-UMIPIC.

	Total (*n* = 2974)	UMIPIC (*n* = 1118)	Non-UMIPIC (*n* = 1856)	*p*
Age (years), mean (SD)	81.2 (7.9)	83.4 (6.9)	79.9 (8.3)	<0.001
Nursing home resident, N (%)	170 (9.2)	67 (6.0)	237 (8.0)	0.002
**Comorbidities**				
Hypertension, N (%)	2663 (89.5)	1029 (92.0)	1634 (88.0)	<0.001
Diabetes mellitus type 2, N (%)	843 (45.4)	518 (46.3)	1361 (45.8)	0.648
Dyslipidemia, N (%)	923 (49.7)	600 (53.7)	1523 (51.2)	0.041
Obesity (BMI > 30), N (%)	1241 (41.7)	461 (41.2)	780 (42.0)	0.673
Atrial fibrillation, N (%)	1027 (55.3)	652 (58.3)	1679 (56.5)	0.118
Ischemic heart disease, N (%)	372 (20.0)	171 (15.3)	543 (18.3)	<0.001
Myocardial infarction, N (%)	533 (17.9)	189 (16.9)	344 (18.5)	0.278
Chronic kidney disease (eGFR< 60 mL/min), N (%)	1798 (60.5)	686 (61.4)	112 (59.9)	0.439
Stroke, N (%)	240 (12.9)	165 (14.8)	405 (13.6)	0.168
Peripheral arterial disease, N (%)	95 (5.1)	61 (5.5)	156 (5.2)	0.734
Dementia, N (%)	126 (6.8)	65 (5.8)	191 (6.4)	0.316
COPD, N (%)	236 (12.7)	126 (11.3)	362 (12.2)	0.248
Neoplasm, N (%)	173 (9.3)	115 (10.3)	288 (9.7)	0.406
Hepatic liver disease, N (%)	14 (0.8)	11 (1.0)	25 (0.8)	0.537
Anemia, N (%)	118 (6.4)	136 (12.2)	254 (8.5)	<0.001
**Clinical Characteristics**				
SBP (mmHg), mean (SD)	139.1 (26.7)	137.4 (25.6)	140.2 (27.4)	0.013
DBP (mmHg), mean (SD)	74.9 (15.8)	73.2 (15.2)	75.9 (16.1)	0.014
HR (lpm), mean (SD)	87.8 (23.0)	84.9 (21.1)	89.5 (23.9)	<0.001
Charlson Index, mean (SD)	2.6 (2.3)	2.9 (2.4)	2.5 (2.2)	0.08
Barthel Index (points), mean (SD)	77.7 (24.4)	76.2 (24.4)	78.6 (24.4)	0.01
Pfeiffer Test, mean (SD)	1.9 (2.2)	1.6 (1.9)	2.1 (2.3)	<0.001
**Laboratory**				
Hemoglobin (g/dL), mean (SD)	11.7 (1.8)	11.6 (1.8)	11.8 (1.8)	0.033
Creatinine (mg/dL), mean (SD)	1.2 (2.3)	1.3 (3.6)	1.2 (0.6)	0.325
eGFR, mean (SD)	55.8 (25.0)	55.7 (25.0)	55.8 (25.0)	0.912
NT- proBNP (pg/mL), median [RIQ]	3430 (6148)	4119 (7070)	3153 (5720)	0.004
Sodium (mEq/L), mean (SD)	138.7 (5.5)	138.3 (4.9)	138.9 (5.9)	0.007
Potassium (mEq/L), mean (SD)	4.3 (0.6)	4.3 (0.6)	4.3 (0.6)	0.051
**Characteristics of cardiopathy**				
LVEF, mean (SD)	55.1 (14.7)	56.3 (14.2)	54.4 (14.9)	<0.001
LVEF > 50%, N (%)	1978 (66.5)	780 (69.8)	1198 (64.5)	0.004
NYHA:				
I	191 (6.5)	37 (3.3)	154 (8.5)	<0.001
II	1580 (54.2)	632 (56.9)	948 (52.5)	
III	1061 (36.4)	411 (37.0)	650 (36.0)	
IV	85 (2.9)	31 (2.8)	54 (3.0)	
**Etiology of HF**				
Hypertensive	1372 (46.1)	600 (53.7)	772 (41.6)	<0.001
Ischemic	543 (18.3)	171 (15.3)	372 (20.2)	<0.001
Valvular	605 (20.3)	196 (17.5)	409 (22.0)	0.003
Unaffiliated	261 (8.9)	70 (6.4)	191 (10.3)	<0.001
Other	193 (6.5)	81 (7.2)	112 (6.0)	0.219
**Treatment**				
Beta blockers, N (%)	2012 (67.7)	781 (69.9)	1231 (66.3)	0.047
ACE inhibitors/ARB, N (%)	1906 (64.1)	687 (61.4)	1219 (65.7)	0.022
Sacubitril valsartan, N (%)	50 (1.7)	21 (1.9)	29 (1.6)	0.557
Aldosterone antagonists, N (%)	591 (19.9)	247 (22.1)	344 (18.5)	0.020
Loop diuretics, N (%)	2214 (74.4)	852 (76.2)	1362 (73.4)	0.091
Thiazide diuretics, N (%)	466 (15.7)	222 (19.9)	244 (13.1)	<0.001
Digoxin, N (%)	727 (24.4)	231 (20.7)	496 (26.7)	<0.001
iSGLT2, N (%)	15 (0.5)	5 (0.4)	10 (0.5)	0.797
Statins, N (%)	868 (29.2)	350 (31.3)	518 (27.9)	0.05
Anticoagulation, N (%)	1017 (34.2)	368 (32.9)	649 (35.0)	0.264
DOAC, N (%)	302 (10.2)	168 (15.1)	133 (7.2)	<0.001
Antiplatelets, N (%)	745 (25.1)	246 (22.0)	499 (26.9)	0.003
Insulin, N (%)	615 (20.7)	232 (20.8)	383 (20.6)	0.963

Abbreviations: UMIPIC: Comprehensive Management Unit for Patients with Heart Failure; ACE inhibitors: Angiotensin-Converting Enzyme Inhibitors; ARB: Angiotensin II Receptor Blocker; BMI: Body Mass Index; COPD: Chronic Obstructive Pulmonary Disease; eGFR: estimated Glomerular Filtration Rate; HR: Heart Rate; LVEF: Left Ventricular Ejection Fraction; NYHA: New York Heart Association; NT-proBNP: N-terminal pro-B-type Natriuretic Peptide; iSGLT2: Sodium–Glucose Co-Transporter 2 Inhibitors; DOAC: Direct Oral Anticoagulant.

**Table 2:** Outcomes of women with heart failure according to care setting.

	Total	UMIPIC	Non-UMIPIC	*p*
Mortality at 30 days, N (%)	149 (6.0)	17 (4.0)	132 (8.0)	<0.01
30-day readmission, N (%)	358 (13.5)	87 (9.0)	271 (18.0)	<0.01
30-day HF readmission, N (%)	259 (10.0)	65 (7.0)	194 (13.0)	<0.01
30-day cardiovascular mortality, N (%)	126 (4.0)	16 (2.0)	110 (6.0)	<0.01
One-year all-cause mortality, N (%)	696 (36.0)	213 (32.0)	483 (40.0)	<0.01
One-year readmission, N (%)	1044 (45.5)	329 (35.0)	715 (56.0)	<0.01
One-year HF readmission, N (%)	700 (36.0)	222 (30.0)	478 (42.0)	<0.01
One-year cardiovascular mortality, N (%)	497 (25.9)	137 (21.9)	360 (30.0)	<0.01

Abbreviations: HF: Heart Failure.

**Table 3:** Univariate and multivariate Cox analysis of risk for 1-year mortality in women.

	Total	UMIPIC	Non-UMIPIC	*p*
UMIPIC	0.790 (0.712–0.875)	<0.001	0.789 (0.713–0.873)	<0.01
Anemia	0.968 (0.814–1.152)	0.716	--	<0.01
Age	1.011 (1.005–1.017)	<0.001	1.011 (1.006–1.017)	<0.01
Nursing home resident	1.144 (0.968–1.353)	0.115	--	<0.01
Hypertension	1.003 (0.858–1.173)	0.967	--	<0.01
Ischemic cardiopathy	1.016 (0.895–1.153)	0.811	--	<0.01
LVEF > 50%	0.946 (0.852–1.051)	0.302	--	<0.01
Beta blockers	0.883 (0.797–0.079)	0.018	0.896 (0.811–0.991)	<0.01

Abbreviations: HR: Hazard Ratio; UMIPIC: Comprehensive Management Unit for Patients with Heart Failure. LVEF: Left Ventricular Ejection Fraction.

**Table 4:** Baseline characteristics of heart failure men from RICA (total) and followed in UMIPIC and non-UMIPIC.

	Total (*n* = 2670)	UMIPIC (*n* = 916)	Non-UMIPIC (*n* = 1754)	*p*
Age (years), mean (SD)	78.0 (9.3)	80.9 (7.8)	76.5 (9.7)	<0.001
Nursing home resident, N (%)	225 (8.4)	43 (4.7)	182 (10.4)	<0.001
**Comorbidities**				
Hypertension, N (%)	2203 (82.5)	788 (**86**.0)	1415 (80.7)	<0.001
Diabetes mellitus type 2, N (%)	1253 (46.9)	426 (46.5)	827 (47.1)	0.775
Dyslipidemia, N (%)	1391 (52.1)	484 (52.8)	907 (51.7)	0.596
Obesity (BMI > 30), N (%)	875 (32.8)	307 (33.5)	568 (32.4)	0.573
Atrial fibrillation, N (%)	1326 (49.7)	490 (**53**.5)	836 (47.7)	0.004
Ischemic heart disease, N (%)	909 (34.0)	284 (31.0)	625 (35.6)	0.018
Myocardial infarction, N (%)	810 (30.3)	277 (30.2)	533 (30.4)	0.965
Chronic kidney disease (eGFR< 60 mL/min), N (%)	1361 (51.0)	490 (53.5)	871 (49.7)	0.061
Stroke, N (%)	381 (14.3)	132 (14.4)	249 (14.2)	0.907
Peripheral arterial disease, N (%)	463 (17.3)	162 (17.7)	301 (17.2)	0.747
Dementia, N (%)	116 (4.3)	32 (3.5)	84 (4.8)	0.134
COPD, N (%)	958 (35.9)	309 (33.7)	649 (37.0)	0.098
Neoplasm, N (%)	380 (14.2)	164 (17.9)	216 (12.3)	<0.001
Hepatic liver disease, N (%)	75 (2.8)	19 (2.1)	56 (3.2)	0.109
Anemia, N (%)	202 (7.6)	96 (10.5)	106 (6.0)	<0.001
**Clinical Characteristics**				
SBP (mmHg), mean (SD)	134.9 (25.9)	131.6 (22.8)	136.7 (27.3)	<0.001
DBP (mmHg), mean (SD)	74.8 (16.5)	72.1 (14.1)	76.2 (16.0)	<0.001
HR (lpm), mean (SD)	84.8 (21.9)	82.0 (20.6)	86.3 (22.4)	<0.001
Charlson Index, mean (SD)	3.5 (2.6)	3.7 (2.6)	3.4 (2.6)	0.019
Barthel Index (points), mean (SD)	86.8 (19.9)	84.8 (20.4)	87.8 (19.5)	<0.001
Pfeiffer Test, mean (SD)	1.2 (1.8)	1.1 (1.4)	1.3 (1.9)	0.008
**Laboratory**				
Hemoglobin (g/dL), mean (SD)	12.4 (2.2)	12.2 (2.1)	12.5 (2.2	0.009
Creatinine (mg/dL), mean (SD)	1.4 (0.7)	1.4 (0.7)	1.4 (0.7)	0.697
eGFR, mean (SD)	62.4 (28.0)	61.8 (27.7)	62.6 (28.2)	0.571
NT- proBNP (pg/mL), median [RIQ]	3969 (6566)	3755 (6485)	4519 (6527)	0.252
Sodium (mEq/L), mean (SD)	138.7 (5.2)	138.6 (5.9)	138.8 (4.7)	0.241
Potassium (mEq/L), mean (SD)	4.4 (0.6)	4.4 (0.6)	4.4 (0.6)	0.782
**Characteristics of cardiopathy**				
LVEF, mean (SD)	47.1 (15.7)	49.3 (14.8)	46.0 (16.1)	<0.001
LVEF > 50%, N (%)	1180 (44.2)	443 (48.4)	737 (42.0)	0.002
NYHA:				
I	232 (8.9)	59 (6.5)	173 (10.2)	<0.01
II	1445 (55.3)	504 (55.2)	941 (55.4)	
III	854 (32.7)	320 (35.0)	534 (31.4)	
IV	81 (3.1)	30 (3.3)	51 (3.0)	
**Etiology of HF**				
Hypertensive	814 (30.5)	343 (37.4)	471 (26.9)	<0.001
Ischemic	909 (34.0)	284 (31.0)	625 (35.6)	0.018
Valvular	321 (12.0)	98 (10.7)	223 (12.7)	0.133
Unaffiliated	284 (10.6)	75 (8.2)	209 (11.-9)	0.003
Other	342 (12.8)	116 (12.7)	226 (12.9)	0.903
**Treatment**				
Beta blockers, N (%)	1855 (69.5)	643 (70.2)	1212 (69.1)	0.565
ACE inhibitors/ARB, N (%)	1648 (61.7)	513 (56.0)	1135 (64.7)	<0.001
Sacubitril valsartan, N (%)	88 (3.7)	33 (3.6)	66 (3.8)	0.914
Aldosterone antagonists N (%)	712 (26.7)	271 (29.6)	441 (25.1)	0.015
Loop diuretics, N (%)	1991 (74.6)	709 (77.4)	1282 (73.1)	0.015
Thiazide diuretics, N (%)	495 (15.2)	184 (20.1)	221 (12.6)	<0.001
Digoxin, N (%)	456 (17.1)	148 (16.2)	308 (17.6)	0.386
iSGLT2, N (%)	19 (0.7)	4 (0.4)	15 (0.9)	0.332
Statins, N (%)	839 (31.4)	306 (33.4)	533 (30.4)	0.114
Anticoagulation, N (%)	820 (30.7)	259 (28.3)	561 (32.0)	0.052
DOAC, N (%)	266 (10.0)	135 (14.7)	131 (7.5)	<0.001
Antiplatelets, N (%)	859 (32.2)	264 (28.8)	595 (33.9)	0.008
Insulin, N (%)	518 (19.4)	170 (18.6)	348 (19.8)	0.440

Abbreviations: UMIPIC: Comprehensive Management Unit for Patients with Heart Failure; ACE inhibitors: Angiotensin-Converting Enzyme Inhibitors; ARB: Angiotensin II Receptor Blocker; BMI: Body Mass Index; COPD: Chronic Obstructive Pulmonary Disease; eGFR: estimated Glomerular Filtration Rate; HR: Heart Rate; LVEF: Left Ventricular Ejection Fraction; NYHA: New York Heart Association; NT-proBNP: N-terminal pro-B-type Natriuretic Peptide; iSGLT2: Sodium–Glucose Co-Transporter 2 Inhibitors; DOAC: Direct Oral Anticoagulant.

**Table 5:** Outcomes of men with heart failure according to care setting.

	Total	UMIPIC	Non-UMIPIC	*p*
Mortality at 30 days, N (%)	142 (6)	19 (2.0)	123 (8.0)	<0.05
30-day readmission, N (%)	317 (13.0)	65 (8.0)	252 (18.0)	<0.01
30-day HF readmission, N (%)	189 (8.0)	39 (5.0)	150 (11.0)	<0.01
30-day cardiovascular mortality, N (%)	101 (3.5)	14 (2.0)	87 (5.0)	<0.001
One-year all-cause mortality, N (%)	699 (39.0)	216 (37.0)	483 (41.0)	<0.05
One-year readmission, N (%)	998 (54.0)	327 (50.0)	671 (58.0)	<0.01
One-year HF readmission, N (%)	542 (33.5)	157 (29.0)	385 (38.0)	<0.01
One-year cardiovascular mortality, N (%)	461 (26.5)	128 (23.0)	333 (30.0)	<0.01

Abbreviations: HF: Heart Failure.

**Table 6:** Univariate and multivariate Cox analysis of risk for 1-year mortality in men.

	Total	UMIPIC	Non-UMIPIC	*p*
UMIPIC	0.782 (0.701–0.873)	<0.001	0.787 (0.707–0.875)	<0.001
Anemia	1.191 (0.995–1.425)	0.057	--	--
Age	0.999 (0.993–1.005)	0.698	--	--
Nursing home resident	0.905 (0.756–1.083)	0.274	--	--
Hypertension	0.983 (0.862–1.121)	0.797	--	--
Atrial Fibrillation	0.945 (0.856–1.044)	0.268	--	--
LVEF > 50%	0.969 (0.867–1.061)	0.414	--	--
Neoplasm	1.159 (1.003–1.320)	0.046	1.153 (1.005–1.322)	0.027
ACE inhibitors/ARB	0.921 (0.831–1.021)	0.117	--	--
Thiazide diuretics	1.174 (1.026–1.342)	0.019	1.162 (1.017–1.328)	0.027
Barthel Index	0.990 (0.988–0.992)	<0.001	0.990 (0.988–0.993)	<0.001

Abbreviations: HR: Hazard Ratio; UMIPIC: Comprehensive Management Unit for Patients with Heart Failure; LVEF: Left Ventricular Ejection Fraction; ACE inhibitors: Angiotensin-Converting Enzyme Inhibitors; ARB: Angiotensin II Receptor Blocker.

## Data Availability

The data presented in this study are available on request from the last author. The data are not publicly available due to privacy restrictions.

## Supplementary Materials

The following supporting information can be downloaded at: https://www.mdpi.com/article/10.3390/jcm14165818/s1. Figures S1 and S2. Kaplan–Meier curve for mortality in woman and men by NYHA functional class.

## Abbreviations

The following abbreviations are used in this manuscript:

UMIPIC	Comprehensive Management Unit for Patients with Heart Failure
RICA	National Registry of Heart Failure
SEMI	Spanish Society of Internal Medicine
NYHA	New York Heart Association
LVEF	Left Ventricular Ejection Fraction
HFpEF	Heart Failure with Preserved Ejection Fraction
DOAC	Direct Oral Anticoagulant
ACEi	Angiotensin-Converting Enzyme Inhibitors
ARBs	Angiotensin Receptor Blockers
