# Supplementary figures and images for: Are There Gender Differences in the Benefits of Multidisciplinary Care in Patients with Heart Failure? Results from the UMIPIC Program
Source: J Clin Med. 2025 Aug 17;14(16):5818. doi: 10.3390/jcm14165818 (PMC12387713; doi:10.3390/jcm14165818)

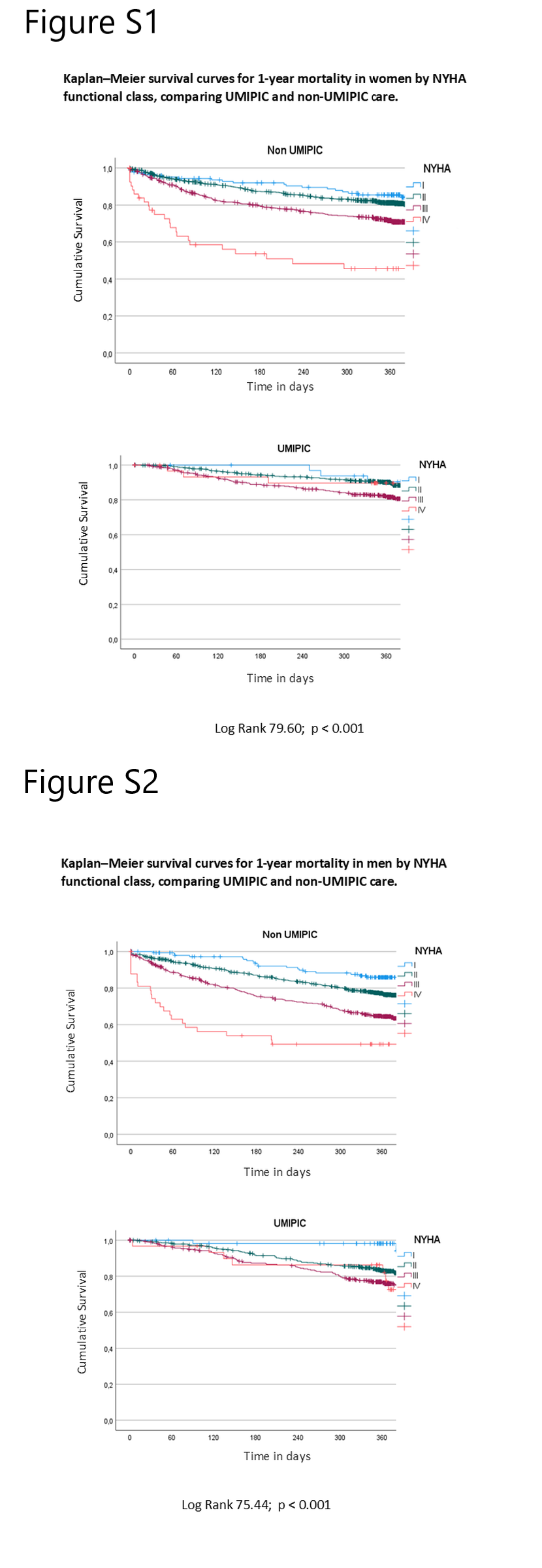

Supplement: Supplementary file 1 [file jcm-14-05818-s001.zip › jcm-3790438-supplementary.png]
